# Welfare Assessment in Equine-Assisted Service (EAS) Horses

**DOI:** 10.3390/ani16101434

**Published:** 2026-05-08

**Authors:** Jéssica Carvalho Seabra, Tanja Hess, Tessa Finnestead, Temple Grandin

**Affiliations:** Department of Animal Sciences, Colorado State University, Fort Collins, CO 80523, USA; tanja.hess@colostate.edu (T.H.); tessa.finnestead@colostate.edu (T.F.); cheryl.miller@colostate.edu (T.G.)

**Keywords:** behavior, therapy, well-being

## Abstract

Equine-Assisted Service (EAS) horses can be exposed to stressors that affect their behavior and welfare. Previous studies investigated behavioral and physiological indicators (PI) only during therapy sessions. This study evaluated the frequency of stress and pain behaviors (FSPB) in horses with different riders, while assessing welfare through housing, management, and PI. Ten horses were observed during EAS sessions with three rider groups: able-bodied, clients with significant physical impairment, and clients with no significant physical impairment, totaling twenty-six riders. Behavioral data was collected using video recordings and analyzed with continuous sampling. Time-budget analysis and PI (ACTH, cortisol, WBC) were evaluated. Blood samples were collected at baseline and after 45 and 90 days working on EASs. There was no difference in FSPB/sec between groups, and the clients’ disability did not seem to affect the variable. Four out of ten horses had high ACTH at baseline and had their medication readjusted, highlighting the importance of periodic blood tests. Individual alterations in immune cell numbers and low cortisol could be associated with chronic stress. Horses spent 49.43% of their time budget eating, suggesting adequate feeding behavior. However, their time lying down was reduced and the effect of strong artificial lights at night needs further investigation.

## Simple Summary

Horses used in equine-assisted services (EAS) can be exposed to stressors that affect their overall well-being. Earlier research mainly focused on therapy session outcomes, without considering other important factors such as general health and management. This study examined how often EAS horses showed stress and pain behaviors while working with different riders, including able-bodied individuals and those with varying levels of physical impairment. Ten horses participated in sessions with 26 riders. Their behavior was recorded with a video camera and analyzed second by second. Researchers also evaluated health indicators through blood tests taken before the study and after 45 and 90 days of work. These tests included hormone levels and immune cell counts. Management and diet were evaluated by analyzing how horses distributed their time during a 24 h period. The results showed that the type of rider did not affect the frequency of stress or pain behaviors. However, blood tests showed that some horses required medication adjustments for pre-existing conditions, which highlights the importance of regular health monitoring. While the horses spent a large portion of time eating, suggesting adequate feeding behavior and natural time distribution, reduced time spent lying down and possible effects of artificial lighting at night need further investigation.

## 1. Introduction

Equine-assisted services (EAS) involve the use of equine-related activities to contribute positively to the cognitive, physical, emotional and social well-being of humans [1,2]. Therapeutic riding programs may involve riders with intellectual disabilities, physical impairments, and emotional or social issues. Horses involved in EAS are working animals, and similarly to those employed for more common equestrian disciplines, they can be subjected to physical and mental stressors that could affect their behavior and welfare levels [3]. As an example, riders in the therapeutic riding programs have different disabilities that can affect their capacity to maintain a balanced position [3] or interfere with the horse–rider communication, negatively affecting the interaction between horses and clients [4]. The lack of balance of a rider is considered a frequent cause of back pain in horses [5]. Previous studies show that pain and stress are frequent causes of hyperreactive and conflict behaviors in horses [6]. At the same time, scientists suggest that chronic pain or chronic discomfort could increase the aggressiveness of horses towards humans [7].

However, it is well recognized that other factors such as housing, diet, and general health conditions can affect equine welfare [8]. For example, recent studies revealed that restricted access to roughage is the main cause for the development of abnormal behaviors in horses [8] and can increase aggression among conspecifics housed in groups [9]. At the same time, researchers observed that horses kept full-time in stables presented modified time-budgets and this parameter, associated with other physiological alterations, indicates that the animals were in a state of chronic stress and poor welfare [10]. “Time budget” refers to how an animal distributes its time in behavioral activities during a period of 24 h. Through direct observation of feral horse populations free in their natural environment, researchers have characterized the natural behavioral pattern of this species. When provided with appropriate conditions, domesticated horses tend to exhibit patterns comparable to those observed in feral individuals, which is why such comparisons are commonly made [11]. In this context, differences between the time budgets of domesticated horses and their feral conspecifics are used as indicators of potential welfare impairment [11,12]. Previous studies were conducted to investigate behavioral and physiological indicators during therapy sessions [1,4,13,14,15], but they did not evaluate the general welfare levels. Factors such as housing conditions, nutritional management and pre-existing health problems can interfere with the relationship between horses and clients, generating imprecise results. Therefore, the goal of the current study was to assess the welfare of equine-assisted therapy horses, considering the interactions between horses and clients, the diet, housing and management conditions, and general health state to identify the main sources of stress.

## 2. Materials and Methods

### 2.1. Animals and Study Design

All experimental procedures were approved by the Institutional Animal Care and Use Committee of Colorado State University (CSU, IACUC protocol number 4729). This observational study took place at The Temple Grandin Equine Center in Fort Collins, CO, USA. It was carried out between January and April 2024, with ten mixed-breed horses (six geldings and four mares). This study included all sound horses used in EAS activities at the facility. At the beginning of the study, the horses were 20.43 ± 1.15 years old (from 17 to 26 years old) and weighed 524.53 ± 23.21 kg, working on average 3 ± 0.31 h per week. The horses were housed in individual pens with iron pipe fencing (7.32 × 17.73 m), and sheds with pine shavings. The horses were kept inside the pens for most of the time, going out only to work in EAS sessions. LED lights were turned on by the facility’s management overnight 10 m away from the pen area for security reasons. A lux meter (LX1330B Light Meter; Dr. Meter Digital Illuminance, Shenzhen, China) was used to measure the intensity of the artificial light inside the horses’ pens at night (0.2 lx). They had unlimited access to mineral blocks and water, were periodically evaluated by a veterinarian and were considered fit for light-intensity exercise during the total duration of the study. The horses were fed to meet requirements for lightly exercising horses [16] (NRC 2007), being fed 2% of BW of hay (Timothy Grass mix) divided into two meals. Commercial senior concentrate was used as a supplement to help horses meet nutritional requirements. The amounts of concentrate offered to each horse varied according to their individual needs. All horses had tactile and visual contact with conspecifics through the fence. The observational study was initiated on the last day of a 21-day break period where the horses were not being used in equine-assisted services or in other types of work (state winter break at the university).

### 2.2. Blood Analyses

Two blood samples (the first one at 8 AM and the second at 4 PM) were collected on the last day of a 21-day break period (baseline). The same procedure was repeated after 45 and 90 days working on Equine-Assisted Services. Blood samples were collected on designated rest days, during which the horses were kept in their pens for a full 24 h period. The sampling was performed by a trained veterinarian inside the animals’ own enclosures. The samples were collected quickly and using positive reinforcement techniques to limit acute stress and thereby prevent any alterations in circulating cortisol levels.

All blood collection procedures were performed during the horses’ day of rest, when animals remained in their pens for 24 h. The procedure took place in the horses’ pen and was conducted by an experienced veterinarian, who collected the samples quickly to minimize acute stress, and consequently to avoid any changes in the blood cortisol levels. The blood samples were drawn by jugular venipuncture into three vacutainers, two with ethylene diamine tetra-acetic acid (EDTA) and one containing heparin.

The first blood sample collected with an EDTA tube was placed on ice and immediately sent to the Veterinary Pathology laboratory at Colorado State University for identification and quantification of the leukogram with differential leukocyte count. The values for the white blood cells (WBCs) were determined via the ADVIA 2120i hematology analyzer (Siemens Healthcare Diagnostics, Erlangen, Germany). Blood smears were done immediately at the university’s laboratory and were used to determine the differential leukocyte counts using a 100-cell count technique. The neutrophil/lymphocyte ratio was calculated by dividing the absolute number of neutrophils by the absolute number of lymphocytes [10].

The second blood sample collected with EDTA was placed on ice and transported to a room inside the TGEC building. The samples were immediately centrifuged at 1500× *g* for 5 min; the plasma was separated, divided into aliquots, and placed into polypropylene tubes and stored at −20 °C. On the following days, the frozen samples were placed in a Styrofoam box containing dry ice and shipped to the Endocrinology Laboratory within the Animal Health Diagnostic Center (AHDC) at Cornell University for ACTH analysis. At Cornell University, the ACTH values were determined by a chemiluminescent immunometric assay (Immulite 2000xpi, Siemens Healthcare, Malvern, PA, USA), and horses with basal plasma ACTH concentrations above seasonally adjusted reference ranges were identified and considered positive for PPID.

The heparinized blood sample was also placed on ice and transported to a room inside the TGEC building. Then, they were immediately centrifuged at 1500× *g* for 5 min, and the plasma was separated, divided into aliquots, and placed into polypropylene tubes and stored at −20 °C. On the following days, the frozen samples were placed in a Styrofoam box containing dry ice and shipped to Cornell University for cortisol analysis. Plasma cortisol levels were measured by chemiluminescent immunometric assay (Immulite 2000xpi, Siemens Healthcare). CCR ratios for each horse were calculated by dividing the difference in the two sample values by the higher one. The CCR ratio was considered abnormal when the result was <0.30 [17]. The absence of a CCR was considered an indicator of chronic stress and poor welfare in horses [18].

### 2.3. Behavioral Assessments During Equine-Assisted Services

For the ridden horse ethogram, four surveillance cameras (Reolink RLC-823S1, Reolink innovation Limited, Hong Kong, China) were fixed to the four corners of the roof beams of an indoor arena. Each horse was filmed during equine-assisted services sessions with an average of three different clients and one experienced able-bodied rider in a simulated equine-assisted services session (control group). The clients involved in this study have been diagnosed with different medical conditions including one or a combination of the following: autism (n = 9); attention-deficit/hyperactivity disorder (ADHD) (n = 3); down syndrome (n = 2); genetic disorder (n = 2); hip replacement/arthritis (n = 1); cerebral palsy (n = 3); intellectual developmental delay (n = 3); heart transplant (n = 1); anxiety (n = 1); blindness (n = 1); speech disorder (n = 2); and emotional disability (n = 1). Each client was filmed four times, and a total of 26 riders were divided into three groups: control (able-bodied riders), clients with no significant physical impairment (NSPI), and clients with significant physical impairment (SPI). Clients were allocated to each group based on two different evaluations. First, the clients’ level of physical impairment was determined by the program coordinator for EAS at TGEC, who is also a certified therapeutic riding instructor with more than 20 years of experience. In the second evaluation, an experienced observer blinded to the type of disability of the riders watched a random part of the footage and classified the rider into either the group with significant physical impairment or the group with no significant physical impairment. The therapeutic riding instructor and the experienced observer had 100% agreement. The sessions were divided into the preparation phase (being groomed and tacked up), the hand-walked phase, and the riding phase. The footage was recorded with a network video recorder or NVR (Reolink RLN8-410, 1TB, Reolink innovation Limited, Hong Kong, China), and the images were downloaded and stored on an external hard drive for further analysis. The video was analyzed with continuous behavioral sampling using the software Reolink Client v8.17.6. The stress-related behaviors observed during interactions between horses and clients were identified using an adaptation of The Ridden Horse Pain Ethogram (Table 1), and all stress behaviors occurring within the recording period of each phase were counted. The data was inserted into a spreadsheet, and the total frequency of pain and stress-related behaviors observed was divided by the total duration of the recording period in seconds [1]. The footage was analyzed by the main researcher, who was an animal scientist specialized in equine behavior with more than nine years of experience with horses. Videos were randomly selected and scored twice by the only observer. Intra-observer reliability for the overall behavior assessment was ≥90% [19].

### 2.4. Time Budget

One camera (AXIS P3719-PLE Network Camera, Axis Communications AB, Mexico City, Mexico) was installed on the fence of a pen at a height of 3 m. Ten horses were randomly selected on different days during the last 10 days working in equine-assisted service sessions, transferred to the monitored pen, and filmed for 24 h with a network video recorder or NVR (AXIS S3008 Recorder 8TB, Axis Communications AB, Warszawa, Poland). Footage was analyzed with continuous behavioral sampling for determination of the horses’ time budgets and used for the evaluation of the housing and management. During the recording day, the observed horse was kept inside the experimental pen for the 24 h of recording. All recorded footage was stored on an external hard drive for subsequent analysis. Continuous behavioral analysis was conducted using the software AXIS Camera Station Client 5 (ACS 5.x). The duration of stereotypic and other abnormal behaviors, as well as additional activities, was quantified on a second-by-second basis. These data were entered into a spreadsheet and used to calculate the animals’ time budgets. Behavioral categories were classified in accordance with an adapted ethogram (Table 2) [9,10,22].

Two observers were involved in the time budget analysis. The footage of eight horses was analyzed by a trained observer, and the rest of the animals were analyzed by the main researcher. The main researcher was an animal scientist with more than nine years of experience with horses, and the trained observer was an undergraduate student in equine sciences with three years of previous experience with horses (amateur rider). The observers in the current study attained ≥90% agreement for all behaviors.

### 2.5. Data Analysis

The horses’ blood parameters (WBC, Cortisol Circadian Rhythm (CCR), ACTH) collected at three different times (on the last day of a 21-day break period (baseline) and after 45 and 90 days working on Equine-Assisted Services) were analyzed in ANOVA using a mixed model with repeated measures. The effect of the three different groups of able-bodied riders (control group), clients with no significant physical impairment (NSPI), and clients with significant physical impairment (SPI) on the total frequency of pain and stress-related behaviors divided by the total duration of the session in seconds was analyzed by ANOVA using a mixed model with repeated measures and applied to the 10 horses with SAS (9.2) software (Cary, NC, Canada). All time-budget data was tabulated and analyzed through descriptive statistics. Absolute and percentage distributions of all observed behaviors were determined, and the maximum and minimum values were presented. Bartlett’s test was used to verify the homogeneity of variances using the statistical program Assistat [24]. The accepted level of significance was *p* < 0.05 and data transformations were not required prior to statistical analysis.

## 3. Results

### 3.1. Blood Analyses

Horses had higher levels of ACTH (*p* = 0.02) at baseline (34.08 ± 4.40 pg/mL) compared to 45 (18.18 ± 4.82 pg/mL) and 90 days (23.25 ± 4.59 pg/mL) working in equine-assisted services. CCR and leucogram were not different (*p* > 0.05). Cortisol levels were higher (*p* = 0.04) at baseline, 2.97 ± 0.18 μg/dL, decreasing to 2.38 ± 0.19 μg/dL at 45 and 2.43 ± 0.19 μg/dL at 90 days. Values of cortisol in the morning were higher than afternoon samples (*p* < 0.0001). The average values of plasma cortisol for blood samples collected in the morning were 3.08 ± 0.15 μg/dL and 2.0893 ± 0.15 μg/dL in the afternoon. The mean ± SD values for ACTH, the leucocyte profile, neutrophil/lymphocyte (N/L) ratio, and cortisol levels can be seen in Table 3.

### 3.2. Behavioral Assessments During Equine-Assisted Services

One hundred and forty hours of video recordings were analyzed with continuous behavioral sampling. The average duration of the riding sessions was 35.33 ± 1.37 min with sessions varying from 9 to 66 min depending on the clients’ needs. There was no difference in the total frequency of pain and stress-related behaviors divided by the total duration of the session in seconds between groups during the riding phase (*p* = 0.35). On average, horses presented a total of 63 stress and pain-related behaviors per riding session. The control rider group had an average of 0.04 ± 0.02 pain and stress-related behaviors/second, the NSPI group had 0.05 ± 0.01, and the SPI group had 0.04 ± 0.01. The NSPI riders had a higher total of repetitions of the behavior, ears turned back during the entire sessions (34.86 ± 3.48) (*p* = 0.04), when compared with the SPI (27.41 ± 4.15), and the control (22.71 ± 16.13). Horses had higher pain and stress-related behaviors/second during the preparation phase 0.08 ± 0.02 (*p* < 0.0001), compared to the phase that the horses were being hand-walked in the arena 0.06 ± 0.02, and the riding phase 0.05 ± 0.02.

The most frequently observed pain and stress-related behaviors during the riding phase were ears rotated back with 3153 observations, followed by tail swishing with 1682 observations and head shake with 892 observations. The least frequently observed pain and stress-related behaviors during the riding phase were head behind vertical with three observations, eyelids closing with three observations and tail clamped with four observations.

### 3.3. Time-Budget

Two hundred and forty-four hours of video recordings were analyzed with continuous behavioral sampling, generating a total of 14,400 observations. On average, horses spent 46.98% ± 3.25 of their time budget eating hay, 2.45% ± 1.10 eating concentrate, 29.27% ± 2.94 standing, 9.97% ± 1.10 observing the surroundings, 1.25% ± 0.51 lying down, and 2% ± 0.83 socializing with neighboring horses. Seven out of ten horses were observed practicing a stereotypy (pawing or headshaking). They spent 0.89 ± 0.64% of the time budget on those behaviors. Horses in the current study spent 1.25 ± 0.51% of their time budget lying down and five of ten horses were not observed lying down within a 24 h period. The percentage of the time budget that horses spent on all observed behaviors is displayed in Table 4.

## 4. Discussion

### 4.1. Blood Analyses

The average age of the horses in the current study was 20.43 ± 1.15 years old, in accordance with prior research showing that experience associated with older age may be a desirable characteristic when selecting EAS horses [26]. On the other hand, aged horses are more susceptible to chronic conditions, which can adversely affect their welfare, indicating the importance of adequate management and preventive health care [27]. Pituitary pars intermedia dysfunction (PPID) is the most common endocrine disorder of geriatric horses and can lead to complications, including laminitis, secondary infections, and metabolic disturbances [28,29]. High levels of basal plasma adrenocorticotropic hormone (ACTH) have been associated with PPID in horses [28]. In the current study, four out of 10 horses had ACTH levels above reference values at baseline. Those individuals were already under treatment for PPID before the study started. They had their medication readjusted after the baseline collection, leading to lower levels of ACTH at 45 and 90 days.

The discrepancies in cortisol values found in the published literature have been attributed to the fragility of the equine cortisol circadian rhythm, which can be affected by minor stressors, environmental factors, and variations between experimental designs [29]. In a previous study [29], researchers observed only a 20% difference between the rhythm peak and nadir of cortisol. They believe that aging in horses could be associated with a loss of adaptability to physiological stressors, leading to decreased cortisol rhythm amplitude. In the current study, the average difference between morning and afternoon samples was superior to 30%; however, 56% of the horses at 45 and 67% at 90 days had cortisol levels below reference values. The lower levels of cortisol could be related to the older age of the horses; however, a study analyzing salivary cortisol [30] and another study analyzing serum cortisol [31] in horses differing in age found no significant effect of age on cortisol. In another study aiming to determine how welfare status is related to cortisol levels in domestic horses found that horses with compromised welfare had lower levels of both fecal cortisol metabolites and plasma cortisol [32]. Therefore, there is a possibility that the lower levels of cortisol in this study could be associated with chronic stress.

The normal range of the neutrophil/lymphocyte ratio for healthy horses is 0.8 to 2.8 [25]. The average values of the current study are within the normal range, but two horses presented values above the reference at baseline. In a previous study investigating a relationship between individual welfare score (as an indicator of the welfare quality) and the neutrophil/lymphocyte ratio in 509 working and 973 reproduction horses, researchers found that the neutrophil/lymphocyte ratio was higher in horses with a poor welfare state [33]. The reference range for total leukocyte values is 5.5 to 10.5 (10^3^/μL) and the average values observed in the current study were within the normal range. However, two horses presented results below the reference range during the baseline and two horses at the 90-day blood collection, indicating that their immunity was decreased. The reference range for neutrophil counts is 3 to 7 (10^3^/μL). The average values observed in the current study are within the normal range, but two horses were below the reference at the 90-day collection. The reference range for lymphocyte counts is 1.5 to 4.0 (10^3^/μL). The average values observed in the current study are within the normal range, but two horses had values below the reference at baseline. In a study investigating how PPID and pergolide treatment affected endocrine and immune function in horses, researchers found that blood cells and lymphocyte counts were reduced in horses with PPID compared to age-matched controls. However, white blood cells and lymphocyte counts were still within normal limits in horses with PPID, even if significantly lower than healthy horses [34]. On the other hand, researchers suggest that alterations in immune cell numbers could be associated with chronic stress and poor welfare in horses [10,35].

### 4.2. Behavioral Assessments During Equine-Assisted Services

There was no difference in the total frequency of pain and stress-related behaviors/seconds between groups during the riding phase. The results found in the current study corroborate previous research that found no significant differences in the mean number of stress-related behaviors among the recreational riders, physically disabled, psychologically disabled, and special education groups [1]. Although horses in the current study presented on average a total of 63 stress and pain-related behaviors per riding session, the frequency of those behaviors does not seem to be directly related to the rider’s presence, absence or type of disability. While being ridden by clients with different disabilities was not considered significantly more stressful than being ridden by able-bodied individuals, the presence of stress and pain-related behaviors during the sessions could be related to health conditions and/or the overall approach of the equestrian property [1].

Horses in the current study had higher pain and stress-related behaviors/second during the preparation phase, which included grooming and tacking up. In an observational study conducted during tacking-up, 97% of horses (total n = 193) showed behavioral alterations like ears back, tail swishing, head tossing, exposure of the sclera, pawing, attempted to bite, and others. The authors concluded that abnormal behavior during tacking-up and mounting was common and could be an anticipation of musculoskeletal pain when ridden [36]. Horses participating in EAS activities are often older horses with prior careers and those anticipatory behaviors could also reflect previous experience, for example, with harsh handling or fear.

### 4.3. Time-Budget

Scientists that conducted studies on horses living in their natural environment observed that this species spent 51 to 63% of their time budget feeding [37]. Horses in the current study spent a total of 49.43% of their time budget eating (46.98% eating hay, and 2.45% eating concentrate), which is close to the behavior of horses free in their natural environment, suggesting good feeding management. However, seven out of 10 horses in the current study were observed practicing a stereotypy (pawing or headshaking). The percentage of the horse population practicing those behaviors is higher than in some previous research [38,39,40,41]. Yet the horses in the current study were observed spending only 0.13 ± 0.06 of their time-budget on those behaviors, which is less than in a previous study where horses kept in stalls and fed large concentrate meals spent about 6% of their time-budget practicing stereotypies [10].

Researchers believe that chronic stress situations early in the animal’s development may be critical to stereotypy predisposition [42] and that once they have been established, it becomes a habit, and it is unlikely to be eradicated [23]. Therefore, the presence of stereotypies in the horse population may reflect previous exposure to chronic stress, and the decreased time spent practicing those behaviors now may suggest improved welfare.

An interesting finding of the current study is the laying down behavior. In previous research carried out at the same institution, polo horses housed in groups spent an average of 102.75 ± 15.19 min lying down [9]. However, this behavior was reduced to an average of 18.05 ± 7.39 min in the current study. In an observational study conducted with 43 horses from three different centers in Poland, researchers found that the time horses spent lying differs significantly between males and females with mares lying down twice as long as stallions and geldings (Mares = 116.6 ± 50.6 min and Males = 62.3 ± 24.3 min) [43]. In the study with polo horses, 86.67% of the group was composed of mares (13 out of 15 animals), while in the current study, only 30% of the observed population was composed of mares (three out of 10 animals). The higher proportion of mares in the polo horse group, when compared to the current study, could have contributed to the higher average number of minutes spent lying down [43,44]. Recumbency is a prerequisite for horses to complete a full sleep cycle [44] and 5 of 10 horses were not observed lying down at all within a 24 h period. Researchers believe that horses need a minimum of 30 min of recumbency per day to achieve rapid eye movement (REM) sleep [44,45]; otherwise, they can suffer with sleep deprivation, which could negatively affect the animals’ welfare and health [44].

In a recent study, researchers investigated the association between REM sleep deprivation and impaired learning during a reversal learning test in 16 healthy horses. They found that horses with shorter REM sleep presented suboptimal mood, performance deficits, and impaired learning [46]. We believe that the lack of REM sleep could potentially influence the frequency of pain and stress-related behaviors during the therapy sessions and this association should be investigated in future studies.

Another factor that could be influencing the lying down behavior in horses is the older age and the presence of orthopedic diseases such as osteoarthritis. However, all horses included in this study were considered fit for light intensity work when the observations occurred. Moreover, a study aiming to assess the influence of chronic orthopedic disease and old age on the time horses lie down found that neither age nor lameness due to chronic orthopedic disease significantly influenced recumbency times in the study [44].

Research suggests that environmental factors can also influence sleep. Based on previous studies, we theorize that overnight artificial lights present in the pens area could have affected the lying down behavior of the horses. According to scientists, exposing a single eye to low-wavelength blue light can cause inhibition of melatonin secretion in the horse [47] and subsequently disturb sleep patterns, decreasing recumbency [44]. A study aiming to determine the effect of environmental factors on equine sleep stages reported that horses exposed to the overnight fluorescent tube lighting expend less time in recumbent sleep stages and increased time in a standing sleep stage [48]. Therefore, we believe that the impact of artificial lighting on equine lying-down behavior requires further study.

Many studies have established protocols to recognize behavioral indicators of stress and pain in horses [10,36,44,49,50]. However, researchers believe that most horse owners and caretakers are still not able to recognize the expression of those behaviors [51,52]. The current study highlights that behavioral observations can be a powerful and affordable tool that will help equine centers to identify underlying health and management problems that may be affecting the animals’ welfare. Authors believe that the creation of educational programs is needed to teach equine owners or handlers how to recognize behavioral patterns that can reflect inadequate management or undetected medical conditions [51].

The current study had some limitations. The small sample size and the fact the study was performed in one single facility can limit the generalizability of the findings. Moreover, some horses had their PPID medication adjusted at the beginning of the study and the interval between blood sampling points could have been too long to detect more short-term variations in the parameters. The lighting was only discovered during the time budget analysis since the facilities are usually not accessed at night. Saddle fit should also be included in future studies for a more complete welfare assessment.

## 5. Conclusions

The study found no difference in the total frequency of pain and stress-related behaviors/seconds between groups during the riding phase. Therefore, being ridden by disabled individuals was no more stressful for horses than being ridden by able-bodied riders (control group). After the baseline blood collection, four out of 10 horses had to have their PPID medication readjusted. This finding highlights the importance of running periodic blood tests in older horses, especially on individuals that are known for having pre-existing health conditions. Some horses had alterations in immune cell numbers and low plasma cortisol that could be associated with chronic stress and poor welfare in horses.

In the current study, the horses’ lying-down behavior was reduced. Overnight artificial lights present in the pens area could have affected the lying down behavior of the horses and this association requires further study.

## Figures and Tables

**Table 1:** Ethogram used for behavioral observation conducted during preparation phase, hand-walked phase, and riding phase [20].

Behavior	Definition
Head Up and Down	Repeated changes in head position (up/down), not in rhythm with the trot
Head tilted	Head tilted or tilting repeatedly
Head in front	Head in front of vertical (>30°) for ≥10 s
Head behind	Head behind vertical (>10°) for ≥10 s
Head toss	Head position changes regularly, tossed or twisted from side to side, corrected constantly
Ears back	Ears rotated back behind vertical or flat (both or one only) ≥5 s; repeatedly lay flat
Eye closing	Eyelids closed, or half closed for 2–5 s; frequent blinking
Sclera	Sclera exposed repeatedly
Mouth opened	Mouth opening shutting repeatedly with separation of teeth, for ≥10 s
Tongue exposed	Tongue exposed, protruding or hanging out, and/or moving in and out repeatedly
Tail clamped	Tail clamped tightly to middle or held to one side
Tail swishing	Tail swishing large movements: repeatedly up and down/side to side/circular; repeatedly during transitions
Gait changes	Spontaneous changes in gait (e.g., breaks from canter to trot, or trot to canter)
Toe drag	Stumbles or trips more than once; repeated bilateral hindlimb toe drag
Stops spontaneously	Reluctance to move forwards (has to be kicked, verbal encouragement), stops spontaneously
Head shake	Repeated rhythmic movement of the head from left to right
Moving backwards	Walking backwards
Bite	Bite movement directed at the rider, leader or side-walker
Peeing	Elimination of liquid excrement
Defecating	Elimination of solid excrement
Pawing	The horse uses its hoof for digging or scratching a surface
Abnormal movements of lips and mouth	Horse performs repetitive tongue, mouth, or jaw movements without any obvious food substrate in the mouth [21]

**Table 2:** Ethogram with description of the horses’ behaviors used for calculation of the animals’ time budgets.

**Normal Behaviors**	
Eat hay	The horse is eating forage
Drink	The horse is looking for water or drinking water
Observes the surroundings	The horse stands near the fence and is observing outside
Stand	The horse is standing inside the pen
Other normal behaviors	Not mentioned in the list above (defecating, lying down, urinating, scratching, walking, rolling over)
**Stereotypies** [23]	
Excessive licking	Animal is licking surfaces without a nutritional purpose
Kicking	Animal is striking against the wall, fence or similar with the hind hoofs
Nodding	Animal is repetitively moving the head up and down
Pawing	Animal is using the hoof for scratching a surface or digging
Box-walking	Animal is walking in circles inside the enclosure
Weaving	Animal is swaying sideways, moving the neck, head, sometimes hindquarters and forequarters
Crib-biting	Animal fixes the incisors teeth on a horizontal surface. The animal then contracts the muscles of the neck, propelling air into the cranial region of the esophagus and producing a distinctive sound
Windsucking	Animal is opening the mouth, contracting the neck muscles and swallowing air, often producing a distinctive sound
**Other Abnormal Behaviors**	
Coprophagy	Animal ingests its own feces
Metal or wood-chewing	Animal chewing on wood or other objects
**Social Behavior**	
Positive social behavior	A horse approaches a conspecific, stops within close proximity and stays in place for a minimum of 5 s, without any aggressive behavior
Negative social behavior	Agonistic behavior characterized by forward extension of the head toward a conspecific, accompanied by ears held in a pinned-back position and the mouth either closed or open

**Table 3:** Mean values and descriptive statistics for the leucocyte profile, neutrophil/lymphocyte ratio, ACTH, cortisol levels and CCR.

	Total Leukocytes (×10^3^/µL)	Neutrophils (×10^3^/µL)	Lymphocytes (×10^3^/µL)	N/L Ratio	ACTH pg/mL	Cortisol (μg/dL)	CCR Ratio
Reference values	5.5–10.5	3–7	1.5–4.0	0.8–2.8 [25]	2–30	2.0–6.0	>0.30
Baseline	6.18 ± 0.33 ns	3.71 ± 0.22 ns	2.30 ± 0.25 ns	1.85 ± 0.26 ns	34.08 ± 4.40 *	2.97 ± 0.18 *	0.91 ± 0.42 ns
45 days	-	-	-	-	18.18 ± 4.82 *	2.38 ± 0.19 *	0.94 ± 0.26 ns
90 days	5.96 ± 0.32 ns	3.24 ± 0.21 ns	2.48 ± 0.19 ns	1.35 ± 0.10 ns	23.25 ± 4.59 *	2.43 ± 0.19 *	1.01 ± 0.35 ns

Numbers with * are significantly different (*p* < 0.05). ns, not significant.

**Table 4:** Percentage of time spent on each observed behavior for 24 h (time budget).

Behaviors	Time (%)	SE ±
Eating hay	46.98	3.25
Standing	29.27	2.94
Observing the surroundings	9.97	1.10
Walking	3.01	0.27
Eating concentrate	2.45	1.10
Sniffing the ground	2.44	0.65
Positive social interaction	1.98	0.83
Lying down	1.25	0.51
Stereotypic behavior	0.89	0.64
Drinking	0.74	0.16
Licking salt	0.35	0.21
Human Interaction	0.20	0.05
Urinating	0.17	0.03
Scratching	0.14	0.03
Defecating	0.12	0.03
Trotting	0.02	0.01
Rolling over	0.02	0.01
Negative social interactions	0.02	0.01
Galloping	0.01	0.01
Kicking	0.01	0.00

## Data Availability

The original contributions presented in this study are included in the article. Further inquiries can be directed to the corresponding author.

## Abbreviations

The following abbreviations are used in this manuscript:

EAS	Equine-Assisted Services
NSPI	No Significant Physical Impairment
SPI	Significant Physical Impairment
WBC	White Blood Cells
CCR	Cortisol Circadian Rhythm
ACTH	Adrenocorticotropic Hormone
N/L	Neutrophil/Lymphocyte
PPID	Pituitary Pars Intermedia Dysfunction
REM	Rapid Eye Movement
